# Investigation of the Possible Correlation between Idiopathic Parkinson's Disease and Diabetes Mellitus in Egyptian Patients: A Pilot Study

**DOI:** 10.1155/2021/2838669

**Published:** 2021-11-12

**Authors:** Afnan AwadAllah Elgnainy, Mohammad Ismail Hamed, Wael Osman Mohamed, Nagwa Ali Sabri

**Affiliations:** ^1^Department of Clinical Pharmacy, Faculty of Pharmacy, Misr University for Science and Technology, Giza, Egypt; ^2^Department of Neurology, Faculty of Medicine, Al-Azhar University, Cairo, Egypt; ^3^Department of Clinical Pharmacy, Faculty of Pharmacy, Ain Shams University, Cairo, Egypt

## Abstract

**Objectives:**

To study the diabetes-Parkinson's disease (PD) linkage.

**Methods:**

The investigators recorded the rapid eye movement sleep behavior disorder screening questionnaire (RBDSQ) score for 60 diabetic patients: 30 patients were treated with metformin-inclusive sulfonylurea and 30 patients were treated with sulphonylurea(s) monotherapy and matched with 30 controls. We evaluated blood glucose kinetics during a 75 g oral glucose tolerance test for (22) nondiabetic parkinsonian patients and (10) controls. The motor complications scores were recorded for all parkinsonian patients using the relevant parts of the Unified Parkinson's Disease Rating Scale (UPDRS) part IV.

**Results:**

Diabetics recorded higher scores of RBDSQ than controls (*p* < 0.001), with no differences related to antidiabetic therapy. In nondiabetic PD patients, after oral glucose, blood glucose was significantly higher at T1 (*p* < 0.001) than controls. Moreover, the total area under the time curve for blood glucose levels was significantly higher in PD compared to controls (281.22 ± 52.25 vs. 245.65 ± 48.63 mg.hr./dL; *p*=0.013). Higher blood glucose levels were associated with motor abnormalities. Diabetic PD patients recorded higher scores of UPDRS (*p* < 0.001).

**Conclusion:**

Diabetes mellitus and Parkinson's disease are linked, which raises concerns about either of them, probably increasing the risk of the other. This trial is registered with NCT03685357.

## 1. Introduction

In reviewing the literature, we found several common pathological and molecular links in both diabetes mellitus (DM) and Parkinson's disease (PD). These pathophysiological pathways include hyperglycemia, insulin resistance, oxidative stress, mitochondrial dysfunctions, inflammation, and misfolded proteins. One or more of these mechanisms may cause the activation of the apoptotic pathways that contribute to neural cell dysfunction and death, which result in the neurological manifestations in PD [1, 2].

Patients suffering from type 2 diabetes mellitus (T2DM) have a higher risk of developing PD [3]. Results from experimental studies revealed that persistent hyperglycemia triggers dopaminergic dysfunction [4]. The presence of DM may also provoke cognitive dysfunction in the elderly [5]. The diagnosis of PD typically occurs when the disease has actually progressed to a relatively late stage in which motor features are clearly evident and significant neurological damage has already happened [6]. Thus, characterization of nonmotor features is potentially beneficial for early identification of the disease [7]. The Movement Disorders Society (MDS) has recently announced the Research Criteria for Prodromal PD [8].

Prodromal PD is well defined as the presence of early signs and symptoms before PD diagnosis is possible [9]. The best described early nonmotor feature of PD is rapid eye movement sleep behavior disorder (RBD), which is quite specific for neurodegeneration, with high conversion ratios; RBD is observed in up to 60% of PD patients [10]. The time between RBD diagnosis and PD onset fluctuates from five to fifteen years, allowing PD prediction [11].

It is worthy to mention that a definitive diagnosis of RBD necessitates a polysomnogram [12]. It is costly and impractical for the general population. Therefore, we assessed the RBD using a questionnaire‐based diagnostic approach. The REM sleep Behavior Disorder Screening Questionnaire (RBDSQ) is extensively used as an effective diagnostic screening tool for RBD in the general population; it can assess several features of RBD including dreams, movements during sleep, sleep quality, and finally, the relationship between dreams and the corresponding actions [13]. Not only DM but also oral antidiabetic agents (OADs) may modify the risk for PD [14]. Metformin is a recognized antidiabetic drug with a high safety profile, a compound that targets both mitochondrial energy assembly and insulin signaling; it has a neuroprotective activity, but its therapeutic use in PD is not yet approved [15].

The reverse causality could be the case. Patients with Parkinson's disease have impaired glucose tolerance more than the age-matched normal population [16]. The supposed mechanism may be the dysautonomia in PD which leads to beta cells dysfunction and insufficient insulin concentrations in response to the elevated PG levels. Age and prolonged inactivity can adversely affect glucose tolerance in patients with parkinsonian syndrome. The influence of L-dopa therapy on glucose metabolism must be taken into consideration since Levodopa and its metabolite dopamine have been shown to cause hyperglycemia [17].

DM was found to affect the clinical presentation and progression of PD [18]. DM has been previously associated with cognitive decline, gait difficulty, and faster motor abnormalities in PD [19]. The hyperglycemic condition caused by T2DM leads to dopaminergic neurons damaged by several signaling pathways [20]. Thus, the effects of levodopa may be reduced in diabetic parkinsonian patients, and in addition, these patients could be at higher risk for the development of motor impairment [21].

The objective of the current study was to evaluate the effect of diabetes mellitus on rapid eye movement sleep behavior disorder as premotor features that may precede Parkinson's disease, the role of combined metformin-sulphonylurea(s) therapy in diabetes mellitus-Parkinson's disease linkage, glucose metabolism in nondiabetic idiopathic parkinsonian (IPD) patients, and the association between blood glucose and levodopa-induced motor complications in IPD patients.

## 2. Methods

### 2.1. Ethics

The present study was approved by the Research Ethics Committee for Experimental and Clinical Studies, Faculty of Pharmacy, Ain-Shams University; approval number (160) and the Neurology department, Faculty of Medicine, Al-Azhar University.

The study was carried out in accordance with the regulations and recommendations of the Declaration of Helsinki (2013), where a written informed consent was obtained from all participants without any obligation to withdraw when they want to.

### 2.2. Design and Subjects

**Figure 1:**
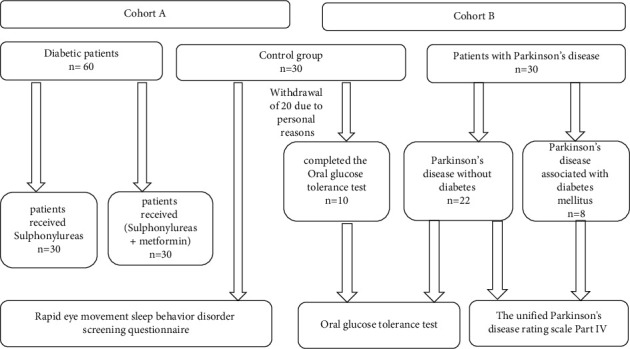
Diagrammatic chart for the study design.

A cross-sectional observational study was conducted in Al-Azhar University Hospital, with 120 participants who were divided into two separate cohorts. The first cohort (Cohort A) included sixty diabetic patients; thirty of them received combined metformin-sulphonylurea(s) therapy, while the others received sulphonylurea(s) monotherapy, and thirty represented the control healthy group. The second cohort (Cohort B) had a total of 40 subjects; thirty of them were parkinsonian patients (22 were diabetic free, while the other 8 were diabetics) and 10 volunteers of the same age and sex, nondiabetic and nonparkinsonian were constituted the control group.  Figure 1 represents the study design.

Inclusion and exclusion criteria are as follows:

- For Cohort A: diabetic patients included were treated with either sulphonylurea(s) monotherapy or combined with metformin with no modification of treatment for a period of three months before assessment.
- For Cohort B: idiopathic Parkinson's disease patients included were diagnosed according to the modified United Kingdom Parkinson Disease Society Brain Bank criteria (2015). Secondary Parkinsonism, drug-induced parkinsonism, and any drugs that might interfere with glucose metabolism were excluded from the study.

### 2.3. Methodology

The participants were subjected to full examination through history taking (age, sex, disease duration, drug history, extended family history, and allergy history). Laboratory data included the estimation of the fasting blood glucose level (FBG) and serum glycated hemoglobin (HbA1c).

#### 2.3.1. Assessment of “Rapid Eye Movement Sleep Behavior Disorder” as a Premotor Feature of Parkinson's Disease in Cohort A

Concerning the sixty diabetic patients, the Rapid eye movement sleep behavior disorder screening questionnaire (RBDSQ) score was recorded in comparison with controls, and then comparison was done between those who received combined metformin-sulphonylurea(s) therapy and sulfonylurea(s) monotherapy. The RBDSQ consists of 10 questions with 13 items overall: items 3, 6.1, 6.2, and 6.3 focus on dream enactment behavior and item 10 asks about central nervous system (CNS) disease. The total score was 13 points, where a score of 5 points was considered the threshold for screening RBD [22].

#### 2.3.2. Blood Glucose Kinetics in Parkinsonian Patients

The kinetics of blood glucose levels during a 75 g oral glucose tolerance test (OGTT) was evaluated for the nondiabetic PD patients (*n* = 22) and controls (*n* = 10). Patients were screened for drugs that might interfere with glucose metabolism, where patients and controls were advised to refrain from smoking thirty minutes before the test, with withdrawal of all antiparkinsonian medications for at least ten hours before the test (only for PD) and fasting overnight.

In addition, glucose values were recorded at fasting (*T*_0_), followed by the oral administration of 75 g of glucose diluted in 250 ml of H_2_O. The patients had to drink the solution within five minutes. The test was discontinued if vomiting had occurred. Then, blood glucose levels were consequently measured at 0.5 hr (*T*_0.5_), 1 hr (*T*_1_), 1.5 hr (*T*_1.5_), and 2 hr. (*T*_2_). The total area under time curve (AUC) mg.hr./dL was calculated according to the trapezoidal method where plasma glucose (PG) levels were measured every 0.5 hours.

The reference PG AUC was calculated as follows:

(1) $$\begin{array}{c} \text{AUC}=\frac{\text{PG}\left(0\right)+2X\text{PG}\left(0.5\right)+2X\text{PG}\left(1\right)+2X\text{PG}\left(1.5\right)+\text{PG}\left(2\right)}{4}. \end{array}$$

#### 2.3.3. Association between Blood Glucose Level and Motor Complications of L-Dopa among Parkinsonian Patients

The unified Parkinson's Disease Rating scale (UPDRS) Part IV was assessed in all PD patients (REF) and a comparison was made between diabetics and diabetic-free parkinsonian patients then a correlation between PG levels and motor abnormalities was done.

### 2.4. Statistical Analysis

#### 2.4.1. Sample Size Calculation

We calculated the required sample size using GPOWER (v.3.1.9.4) and R (v.3.6.1) software (package PWR v.1.2.2). We aimed at detecting a moderate to high effect (*w* = 0.4) of metformin on positive RBDSQ test scores. Using the Chi-square test, a desired power level of at least 80%, and a 5% significance level with 1 degree of freedom, the required sample size was 49 subjects. We, therefore, recruited 60 patients for the expected dropouts or failures in score processing.

Concerning the IPD, expecting a weak-to-moderate effect (Cohen's *f* = 0.20), using a two-way ANOVA assessing in-between subjects' factor interaction, a desired power level of at least 80%, a 5% significance level, assuming sphericity a priori (*ε* = 1), the total sample size required was 32 subjects; 2 patients died before completing the study.

#### 2.4.2. Recorded Data Were Analyzed Using the Statistical Package for the Social Sciences (SPSS Inc., Chicago, Illinois, USA)

Recorded data were analyzed using the statistical package for the social sciences, version 23.0 (SPSS Inc., Chicago, Illinois, USA). The quantitative data were presented as mean ± standard deviation and ranges when their distribution was parametric (normal), while nonnormally distributed variables (nonparametric data) were presented as median with the interquartile range (IQR). Also, qualitative variables were presented as number and percentages. Data were explored for normality using the Kolmogorov–Smirnov and Shapiro–Wilk tests.

The following tests were performed: independent-sample *t*-test of significance was used when comparing between two means; one-way analysis of variance (ANOVA) was used when comparing between more than two means; post hoc test: least significant difference (LSD) was used for multiple comparisons between different variables; two-way ANOVA was used to study the effect of different tested variables and their interaction; the Mann–Whitney *U* test was performed for two-group comparisons in nonparametric data; the Kruskal–Wallis test was performed for multiple group comparisons in nonparametric data; and the comparison between groups on qualitative data was done using the chi-square test and Fisher's exact test instead of the chi-square test only when the expected count in any cell was less than 5.

Pearson's correlation was performed to evaluate the correlation between HbA1c and RBDSQ scores/glucose level (FBG-HbA1c) and motor abnormalities. Multivariate logistic regression analysis: odds ratios (OR) with 95% confidence intervals were computed to assess the overall association between each possible factor affected RBDSQ & UPDRS. The confidence interval was set to 95% and the margin of error accepted was set to 5%. Therefore, the *p* value was considered significant as the following *p* value <0.05 was considered significant.

## 3. Results

### 3.1. Demographic Data

- For Cohort A: there were no significant differences between groups for demographic data, while hypertension was significantly higher in diabetics than controls
- For Cohort B: there were no significant differences between groups for demographic data, while hypertension was significantly higher in PD patients than controls

The demographic data of participants is presented in Table 1 and Table 2.

### 3.2. Main Outcomes

#### 3.2.1. Regarding Cohort A

**Figure 2:**
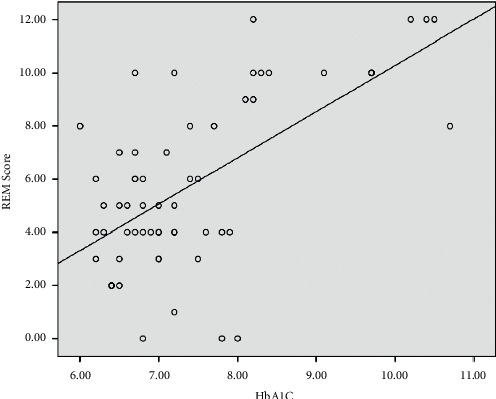
Positive and significant correlation between the RBDSQ score and HbA1c.

- The RBDSQ score was recorded in 30 diabetics who received sulphonylurea(s) monotherapy and another 30 patient who received sulphonylurea(s) combined with metformin in comparison with 30 controls. The RBDSQ score presented as the median (IQR) gave the following results: the “sulphonylurea(s) + metformin group” 5.5 (6), sulphonylurea(s) group 5 (6), and control 2 (4). Analysis of the data using Kruskal–Wallis-H revealed that there is a statistically significant difference between the groups (*p* < 0.001), where *p* value of the Kruskal–Wallis test was <0.05 and Mann–Whitney tests (corrected with Bonferroni's method for multiple comparisons) were conducted to assess pairwise relationships. The results revealed that the RBDSQ were significantly higher in both diabetic groups than the control group, while no difference was found between the (sulphonylurea(s) + metformin) group and the (sulphonylurea(s)) group.
- Using Pearson's correlation, a positive and significant association between the glucose level and RBDSQ score is presented in Figure 2.
- Logistic regression analysis of factors affected the RBDSQ in diabetic patients revealed that age (years) and HbA1c have a significant effect on the RBDSQ, while the other factors are insignificant as seen in Tables 3 and 4.

#### 3.2.2. Regarding Cohort B

**Figure 3:**
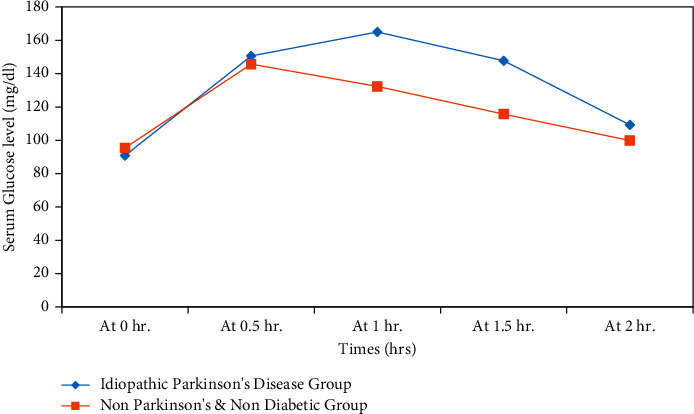
Blood glucose kinetics during the 75 g oral glucose tolerance test (OGTT).

*(1) Blood Glucose Kinetics after 75 g OGTT in Diabetes-Free Parkinsonian Patients*. Analysis of the data using a two-way repeated-measures ANOVA showed that the blood glucose level was significantly higher in (22) nondiabetic PD patients at T1 (*p* < 0.01) compared to (10) controls. Moreover, the total (AUC) for blood glucose levels was significantly higher in PD patients (281.22 ± 52.25) compared to controls (245.65 ± 48.63) mg.hr./dl; (*p*=0.013). The data are shown in Figure 3.

*(2) Association between the Glucose Level and Motor Complications of L-Dopa among IPD Patients*.

**Figure 4:**
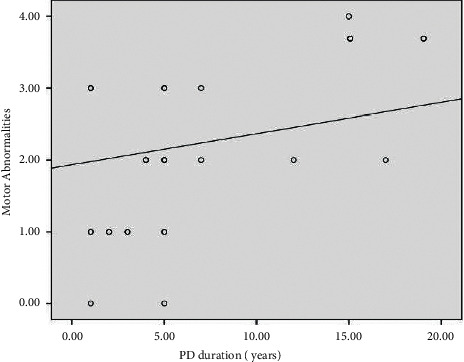
Scatter plot between motor abnormalities and PD duration.

**Figure 5:**
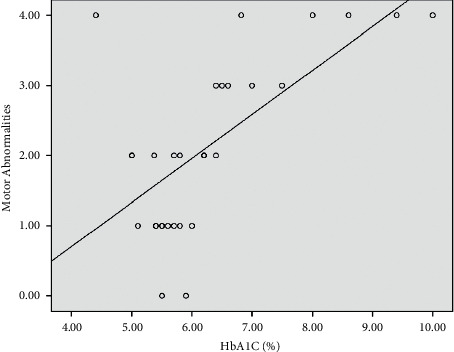
Scatter plot between motor abnormalities and HbA1c.

**Figure 6:**
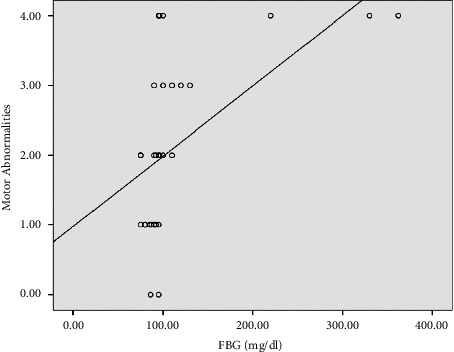
Scatter plot between motor abnormalities and FBG.

- Motor abnormality scores presented as median (IQR) gave the following values: idiopathic Parkinson's disease patients (*n* = 22) recorded as 1.5 (1), while diabetic idiopathic Parkinson's disease patients (*n* = 8) recorded as 4 (1). Analyzing the data by the Mann–Whitney *U* test, the results showed a statistically significant difference between subgroups according to motor abnormalities (*p* < 0.001).
- Positive and significant association between the glucose level and motor abnormalities is presented in Figures 4–6. Using the Pearson correlation coefficient, the results show a positive and significant correlation between motor abnormalities with Parkinson's disease duration, fasting plasma glucose (FPG), and HbA1c.
- Logistic regression analysis of the factors affecting the motor complications (as indicated by the UPDRS) shows that smoking and HbA1c have a significant effect on the UPDRS, while the others are insignificant, *p* < 0.05, as seen in Table 5.

## 4. Discussion

Much epidemiological evidence supports the positive association between DM and PD risk [23, 24]. The shared mechanisms in the pathophysiology of both diseases include hyperglycemia, mitochondrial dysfunction, oxidative stress, and inflammation.

In addition to the previous mechanisms, some miRNAs play an important role in cell differentiation, regulation of the cell cycle, and apoptosis. These miRNAs mediate the insulin pathway, glucose absorption, and PD-related genes. Therefore, studying the common miRNA biomarkers of both PD and DM can explain how these two diseases are correlated, and targeting miRNAs may have a therapeutic value [25].

Generally, genetic susceptibility, lifestyle choices, and exposure to toxic environmental factors can lead to mitochondrial dysfunction, endoplasmic reticulum (ER) stress, inflammation, and metabolic dysregulation. The dysregulation of these pathways may lead to neurodegenerative disorders and/or diabetes. So, we can ensure that PD and DM, both chronic diseases related to age, share similar dysregulated pathways [26].

In the present research, numerous lines of evidence for a correlation between idiopathic Parkinson's disease (IPD) and DM were found, including the following: (a) Diabetes was linked to the probable REM sleep behavior disorder (pRBD). However, OADs did not modify this risk, patients who received a combination of sulphonylurea(s) and metformin recorded similar risk scores to those who received sulphonylurea(s) monotherapy. (b) Parkinsonian patients had impaired glucose metabolism during the OGTT. (c) Moreover, the results showed that diabetic parkinsonian patients recorded higher UPDRS scores.

It would be also of interest to interpret these findings in the context of Egyptian population.

It appears that there are geographic and ethnic differences in the clinical manifestations, epidemiology, and mortality of PD [27]. The effect of comorbidities on PD risk is an important existing topic in research. For example, type 2 diabetes mellitus (T2DM) has been shown to be associated with subsequent PD [23]. South Asians are known to have an increased risk of T2DM which is somewhat determined by genetic factors in addition to diet and lifestyle [28].

Previous studies comparing European PD patients to African PD patients revealed that patients in Africa have more severe disease, but despite this, are taking lower doses of levodopa [29, 30].

It is worthy to mention that no previous Egyptian studies previously discussed a prospective association. However, a previous Egyptian study focused only on postural stability in parkinsonian patients versus those with type 2 diabetes mellitus. These assumptions suggest that diabetes mellitus worsens the features of Parkinson's disease [31]. This study revealed that diabetes mellitus may be a risk factor for Parkinson's disease and vice versa; Parkinson's disease may trigger a glucose metabolism dysfunction, which, in turn, may be a nonmotor feature of PD. Although most previous studies [23, 24] depended on patients' medical history, the present study can predict future risks that may interfere with a patient's quality of life.

In the present study, the diabetic patients recorded higher scores of the RBDSQ, which reflects a higher risk for premotor symptoms; patients who received combined metformin-sulphonylurea(s) failed to modify the risk of premotor symptoms compared with those who received sulphonylurea(s) monotherapy. These findings were in accordance with that reported by Wahlqvist et al., where the incidence of PD risk increased 2.2-fold in T2DM patients [32].

It was found that metformin can beneficially affect neurodegenerative disorders, where the suggested mechanism is that metformin, which causes sensitization to insulin, apart from diabetes treatment, can affect serum lipid profiles, possesses an anti-inflammatory, antiapoptotic action, and antioxidative properties [33, 34].

However, it was reported that antidiabetic drugs such as metformin, sulphonylurea(s), and thiazolidinediones (rosiglitazone and pioglitazone) failed to reduce the risk of restoring the observed impairment in learning and cognition probably because of their: rapid degradation, poor penetrance of the blood brain barrier, and inability to reduce insulin resistance in vivo [35].

In addition, the 75 g (OGTT) gives a prediction for future diabetes risk in addition to being considered the gold standard for diagnosing DM [36], the results supported the concept of impaired glucose metabolism in PD patients. The blood glucose kinetics curve in the current study showed a statistically significant increase in the value of blood glucose at 1 hr in comparison with the corresponding control group, indicating a risk of future diabetes in accordance with the American Diabetic association [12].

Current findings are consistent with those from a previous study that demonstrated the dysregulation of blood glucose in PD patients following an OGTT [16]. Supposed mechanisms could include excessive endogenous glucose production (EGP) and/or insufficient glucose uptake [37]. Impaired glucose metabolism detected in PD patients might be due to failure of the beta cells to secrete insulin in response to the elevated blood glucose. Both beta cell proliferation and function are controlled by the autonomic system [38]. Thus, dysautonomia in PD may lead to beta cells dysfunction and insufficient insulin concentrations in response to the elevated PG levels.

In the current study, higher scores of levodopa-induced motor complications were associated with diabetes mellitus, in agreement with other previous studies which confirmed this association [21, 39]. The proposed mechanism is that chronic dopamine stimulation has been suggested to trigger levodopa-induced motor complications [40]. Chronic hyperglycemia diminishes striatal dopaminergic activity in mice [39], which may facilitate the development of levodopa-induced motor complications in diabetic parkinsonian patients [21]. Thus, diabetes mellitus may be an additional risk factor for levodopa-induced motor complications in PD patients [41].

## 5. Conclusion

From the results obtained in the current study, it can be concluded that diabetic patients have higher REM sleep behavior disorder scores which may be a premotor feature of Parkinson's disease and combined metformin-sulphonylurea(s) did not modify this risk in comparison with sulphonylurea(s) monotherapy. Glucose metabolism is impaired in PD patients, which may be a nonmotor complication of PD. Finally, diabetes negatively affects levodopa induced motor complications.

## Figures and Tables

**Table 1:** Demographic data of the diabetic patients and controls.

	Control (*n* = 30)	SU (*n* = 30)	SU + Met (*n* = 30)	*p*
Age (years)				
Median (range)	59.5 (53–80)	59 (50–85)	57.5 (52–90)	0.77^1^
Gender				
Female	14 (46.7%)	8 (26.7%)	7 (23.3%)	0.11^2^
Male	16 (53.3%)	22 (73.3%)	23 (76.7%)	
Smoking history				
Nonsmoker	24 (80.0%)	17 (56.7%)	16 (53.3%)	0.065^2^
Smoker	6 (20.0%)	13 (43.3%)	14 (46.7%)	
Hypertension				
No	22 (73.3%)	11 (36.7%)	19 (63.3%)	0.01^2^
Yes	8 (26.7%)	19 (63.3%)	11 (36.7%)	
HbA1c (%)				
Median (range)	5.6 (5.0–6.4)	7.2 (6.2–10.5)	7.0 (6.0–10.7)	<0.001^1^
DM duration (years)				
Mean ± SD (range)	—	11.50 ± 6.04 (5–20)	12.83 ± 6.76 (2–25)	0.424^4^
RBDSQ score				
Median (range)	2 (0–7)	5 (3–12)	5.5 (0–12)	<0.001^1^
RBDSQ positive^*∗*^				
Negative	26 (86.7%)	13 (43.3%)	13 (43.3%)	<0.001^3^
Positive	4 (13.3%)	17 (56.7%)	17 (56.7%)	

^1^Kruskal–Wallis rank sum test; ^2^Pearson's chi-square test; ^3^Fisher's exact test for count data; ^4^Student's *t*-test. DM: diabetes mellitus; Met: metformin; RBDSQ: REM sleep Behavior Disorder Screening Questionnaire; SU: sulphonylurea.

**Table 2:** Demographic data of the IPD study subjects with/without DM versus the control group.

	Controls (*n* = 10)	IPD (*n* = 22)	DM + IPD (*n* = 8)	*p*
Age (years)				
Mean (SD)	62.5 (7.79)	64.77 (9.7)	64.25 (10.09)	0.82^1^
Gender				
Female	5 (50.0%)	6 (27.3%)	6 (75.0%)	0.06^2^
Male	5 (50.0%)	16 (72.7%)	2 (25.0%)	
Hypertension				
No	8 (80.0%)	8 (36.4%)	1 (12.5%)	0.01^2^
Yes	2 (20.0%)	14 (63.6%)	7 (87.5%)	
Smoking history				
Nonsmoker	7 (70.0%)	10 (45.5%)	7 (87.5%)	0.11^2^
Smoker	3 (30.0%)	12 (54.5%)	1 (12.5%)	
HbA1c (%)				
Mean (SD)	5.51 (0.38)	5.66 (0.53)	7.98 (1.27)	<0.001^1^
UPDRS scores				
Median (range)	—	1.5 (0–4)	4 (3–4)	<0.001^3^

^1^Linear model ANOVA; ^2^Fisher's exact test; ^3^Mann–Whitney *U* test. DM: diabetes mellitus; IPD: idiopathic Parkinson's disease; UPDRS: Unified Parkinson's Disease Rating Scale.

**Table 3:** Logistic regression analysis of factors affected the RBDSQ in diabetic patients.

Factors	B	SE	Wald	Sig.	Odds ratio	95% CI	
						Lower	Upper
Age (years)	0.147	0.062	5.621	0.018^*∗*^	1.158	1.026	1.307
Sex	0.528	0.930	0.322	0.570	1.695	0.274	10.499
Smoking	1.003	0.938	1.143	0.285	2.727	0.434	17.149
DM duration	−0.116	0.082	1.974	0.160	0.891	0.758	1.047
HTN	−0.376	0.782	0.231	0.631	0.686	0.148	3.181
HbA1c	1.137	0.408	7.762	0.005^*∗*^	3.117	1.401	6.935

B: regression coefficient; SE: standard error; CI: confidence interval. *p* value >0.05 NS; ^*∗*^*p* value <0.05 S; ^∗∗^*P* value <0.001 HS.

**Table 4:** Logistic regression analysis of factors affected the RBDSQ in diabetic patients and control cases.

Factors	B	SE	Wald	Sig.	Odds ratio	95% CI	
						Lower	Upper
Age (years)	0.096	0.039	6.115	0.013^*∗*^	1.101	1.020	1.189
Sex	−0.111	0.773	0.021	0.885	0.895	0.197	4.071
Smoking	0.914	0.800	1.306	0.253	2.494	0.520	11.952
DM duration	−0.036	0.054	0.436	0.509	0.965	0.868	1.073
HTN	0.552	0.649	0.722	0.395	1.737	0.486	6.202
HbA1c	1.026	0.279	13.530	<0.001^*∗*^	2.789	1.615	4.817

B: regression coefficient; SE: standard error; CI: confidence interval. *p* value >0.05 NS; ^*∗*^*p* value <0.05 S; ^∗∗^*P* value <0.001 HS.

**Table 5:** Logistic regression analysis of factors affecting of UPDRS in parkinsonian patients.

Factors	B	SE	Wald	Sig.	Odds ratio	95% CI	
						Lower	Upper
Sex	−0.448	1.751	0.065	0.798	0.639	0.021	19.776
Age (years)	0.125	0.100	1.564	0.211	1.133	0.932	1.377
HTN	−3.545	2.698	1.726	0.189	0.029	0.000	5.719
Smoking	−3.282	2.972	1.220	0.043^*∗*^	6.813	1.356	11.780
HbA1c	2.382	1.140	4.366	0.037^*∗*^	5.823	1.159	10.068

B: regression coefficient; SE: standard error; CI: confidence interval. *p* value >0.05 NS; ^*∗*^*p* value <0.05 S.

## Data Availability

The data used to support the findings of this study are available from the corresponding author upon request.

## Abbreviations

AUC: Area under time curve
DM: Diabetes mellitus
EGP: Endogenous glucose production
FBG: Fasting blood glucose level
HbA1c: Serum glycated hemoglobin
IPD: Idiopathic Parkinson's disease
IQR: Interquartile range
MDS: Movement disorders society
OADs: Oral antidiabetic agents
OGTT: Oral glucose tolerance test
PD: Parkinson's disease
PG: Plasma glucose
pRBD: Probable REM sleep behavior disorder
RBD: REM sleep behavior disorder
RBDSQ: REM sleep Behavior Disorder Screening Questionnaire
T2DM: Type 2 diabetes mellitus
UPDRS: Unified Parkinson's Disease Rating Scale.
